# Effects of Exposure to Different Types of Microplastics on the Growth and Development of *Rana zhenhaiensis* Tadpoles

**DOI:** 10.3390/toxics13030165

**Published:** 2025-02-26

**Authors:** Shimin Xiao, Hao Chen, Xiyao Gao, Xinni He, Rongzhou Jin, Yunqi Wei, Shuran Li, Lei Xie, Yongpu Zhang

**Affiliations:** 1 College of Life and Environmental Science, Wenzhou University, Wenzhou 325035, China; 2 College of Life Sciences, Zhejiang Normal University, Jinhua 321004, China; 3 Zhejiang Key Laboratory of Medical Epigenetics, School of Basic Medical Sciences, Hangzhou Normal University, Hangzhou 311121, China; 4 Herpetological Research Center, College of Life Sciences, Nanjing Normal University, Nanjing 210023, China; 5 School of Life Sciences, South China Normal University, Guangzhou 510631, China; 6 Wenzhou No.30 Middle School, Wenzhou 325019, China; 7 Zhejiang Provincial Key Laboratory for Water Environment and Marine Biological Resources Protection, Wenzhou University, Wenzhou 325035, China

**Keywords:** microplastic, *Rana zhenhaiensis*, growth and development, toxicology

## Abstract

Microplastic (MP) pollution is a major environmental problem, but a comparative study of the toxicological effects of different MPs remains lacking. To explore the toxicological effects of three different microplastics, namely, polypropylene (PP), polystyrene (PS) and polyethylene (PE), Zhenhai brown frog (*Rana zhenhaiensis*) tadpoles were used as the model animal. The results showed that exposure to PE and PS significantly reduced the metamorphosis rate of the tadpoles. Compared with the control group, the body weight of tadpoles in all MP treatments was significantly reduced compared with that of the control group. In addition, exposure to PE reduced the body length and hind limb length of tadpoles. The number of pigment cells increased and intercellular spaces expanded in the liver tissues of tadpoles receiving PS and PE treatments. The composition and function of the intestinal microbiota in the PP treatment and control groups were similar, whereas between the PS treatment and control, they differed. Liver transcriptome sequencing revealed significant alterations in key genes associated with oxidative stress, energy metabolism, immune response, and apoptosis signaling pathways with PS treatment and PP treatment. In summary, MPs may have harmed tadpoles to varying degrees by interfering with related signaling pathways. The negative effects of PE and PS were greater than those of PP.

## 1. Introduction

Microplastics (MPs) are often referred to as plastic particles less than 5 mm in diameter [1]. There are two main sources of MPs in the environment: (1) primary microplastics, which are virgin MPs when produced, such as microbeads in cosmetics and plastic pellets for manufacturing; and (2) secondary microplastics, derived from environmental fragmentation of plastic waste [2]. Based on their chemical composition, the many types of MPs can be divided into categories such as polypropylene (PP), polyethylene terephthalate (PET), polystyrene (PS), polyethylene (PE), polyvinyl chloride (PVC), and other typical types [3]. MPs have stable physical and chemical properties and are difficult to degrade in the natural environment. Because of their light weight, small size, and ability to drift easily, they can easily migrate into any environmental medium. Through river transfer and direct discharge accumulation, MPs eventually accumulate in water bodies [4], rendering MP pollution an urgent and critical environmental issue on a global scale [5].

MPs are commonly found in marine and freshwater environments around the world [6]. In marine environments, MPs are transported long distances by ocean currents and have been found in waters [7,8], as well as in polar regions and deep-sea sediments [9]. In freshwater, MPs can also be detected globally including in large lakes, rivers, and small lakes, ponds, streams, ditches, and springs [10]. The concentration of MPs in the water can reach up to 930 items/L in Yellow River [11,12], and 265 particles/100 mL in Krukut River [13]. Among them, PS, PE, PP, and PET are the most prevalent types of MPs in aquaculture environments and rivers [14,15]. Therefore, in the context of widespread MP pollution, it is of great practical significance to carry out research on the biotoxicological effects of MPs [16].

An abundance of studies have shown that MPs can cause various negative effects, including impaired growth, neurotoxicity, reproductive toxicity, immunotoxicity, oxidative stress, metabolic disturbances, and histopathological alterations in aquatic organisms [17]. For example, exposure of Javanese medaka fish (*Oryzias javanicus*) to PS MPs can cause significant inflammation and tissue damage in intestine, liver, and kidney tissues and can trigger a series of neurotoxic responses characterized by induced oxidative stress, lipid peroxidation, and inhibition in its brain [18]. PP MPs inhibit the glycolysis/gluconeogenesis and oxidative phosphorylation pathways in zebrafish (*Danio rerio*), resulting in the disruption of mitochondrial energy metabolism and behavioral disorders [19]. Additionally, PE MPs accumulate in the gills, gastrointestinal tract, liver, and blood of Barker frog (*Physiaemus cuvieri*) tadpoles, adversely affecting tissues [20]. Exposure to PET MPs increased mortality and the Redox Balance Index while also inducing neurochemical dysfunctions in *P. cuvieri* tadpoles. Interestingly, most adverse effects manifested specifically after the exposure period, indicating a legacy effect of MPs [21]. However, existing laboratory studies primarily focus on the toxicological effects of individual MPs or their combined effects but lack direct comparative research on the differential impacts of various MP types within the same species.

In this study, the aim was to investigate the differences in the toxicological effects of different MP types. Based on their environmental prevalence and commercial accessibility, PP, PE, and PS were selected as the experimental materials. In addition, the Zhenhai brown frog (*Rana zhenhaiensis*), a common amphibian belonging to the Ranidae and *Rana* genera in eastern and southern China, is suitable for ecotoxicological studies [22] and has been used as a model in our previous study [23]. Thus, in this study, *R. zhenhaiensis* tadpoles were used to explore the different effects of PP, PE, and PS on their growth and development, intestinal microbiota, and histopathology and transcriptome in the liver. Here, we evaluated whether exposure to different MPs affects mortality, metamorphosis, morphological indicators, liver histopathology, and the intestinal microbiota compared to control conditions, and whether molecular mechanisms associated with oxidative stress, energy metabolism, immune response, and apoptosis signaling pathways in the liver could explain the observed differences in growth and development after the exposure period. The results of this study will further explore the environmental hazards of the three kinds of MPs and the possible molecular toxicity mechanism and provide a basis for the ecological protection of amphibians.

## 2. Materials and Methods

### 2.1. Animals and Experimental Design

Eggs of *R. zhenhaiensis* were collected from streams and rivers around Daluo Mountain (27°90′62.94″ E, 120°71′46.37″ N), Wenzhou City, Zhejiang Province, China, in March 2021. The background concentration of MPs was 4.39 ± 1.13 items/L at the sample site [23]. The eggs were carefully moved to portable fish cages filled with water and returned to the laboratory, where they were incubated at normal room temperature using dechlorinated tap water. PP, PS, and PE MPs were purchased from Huachuang Plastic Raw Materials Firm, Dongguan, China. According to the product specifications, these raw MPs are spherical with a diameter of 6 μm, exhibiting high uniformity in particle size distribution and high purity.

According to studies reviewed by Koelmans et al. [12], the MP concentrations in aquatic environments typically range from 1 × 10^−2^ to 10^8^ particles/m^3^. For small water bodies that serve as primary habitats for tadpoles, the MP concentrations range from 0.48 to 21.52 particles/L [11]. Based on related studies [20,24,25], the exposure concentration of MPs in the present study was maintained at 50 mg/L, corresponding to 3.53 × 10^−6^ particles/m^3^—a range potentially observable in severely contaminated freshwater systems.

The solution was prepared according to the following procedure: a 0.05 g sample of PP, PS, or PE microsphere powder was placed into 100 mL of deionized water, and the resulting solution (500 mg/L) was placed into an ultrasonic instrument (Kunshan, KQ-300VDV) (45 Hz) and mixed for 30 min. Stock solutions were then diluted with dechlorinated tap water to obtain target concentrations of 50 mg/L PP, PS, or PE.

The experiment was initiated when tadpoles reached Gosner stage (Gs) 26 [26], at which stage they swam freely and began to feed. Tadpoles were randomly selected and allocated into four experimental groups: control group with dechlorinated tap water only and 50 mg/L PP-, 50 mg/L PS, and 50 mg/L PE-treated groups. Exposure was performed in glass containers (60 × 30 × 15 cm) containing 4 L of dechlorinated tap water or treatment solution. Each container housed 40 tadpoles, resulting in a total of 120 individuals per treatment group. Experiments were performed at approximately 15 ± 2 °C, with a light-dark cycle of 12 h:12 h. Spirulina and cooked fresh lettuce were provided in sufficient quantities daily. Dead tadpoles were promptly removed upon detection, and the cumulative mortality rate was calculated by the mortality rate (%) = (number of cumulative dead tadpoles at the end of exposure/initial number of tadpoles) × 100. To ensure water quality, 2/3 of the test solution was renewed every 2 d, and a multi-function water quality detector (AZ86031) was used to detect dissolved oxygen, pH, conductivity, solid solubility, and salinity. The exposure time ended when the tadpoles reached Gs 42, at which stage their forelimbs emerged and they had the ability to leave aquatic environments for terrestrial locomotion.

### 2.2. Measurement of Morphological Indicators

Thirty tadpoles at Gs 42 were randomly collected from each treatment (n = 30/treatment), anesthetized with 1% MS-222, blot-dried, and weighed with an electronic balance (0.0001 g). Their body length and hind limb length were measured by digital vernier calipers (0.01 mm) according to the guidelines reported by Jiang and Li (2021) [27]. In addition, the cumulative metamorphosis rate for each group was calculated by recording the cumulative number of tadpoles that reached Gs 42 daily. The rate was determined using the following formula: metamorphosis rate (%) = (number of cumulative metamorphosis tadpoles/initial number of tadpoles) × 100.

### 2.3. Liver Histopathology

Three tadpoles per treatment (n = 3/treatment) at Gs 42 were collected and anesthetized (MS-222) for liver histopathology. After being anesthetized, the intact liver was dissected, removed, fixed in 4% paraformaldehyde for 24 h, embedded in paraffin, and then sectioned to 5 μm. After staining the sections with hematoxylin and eosin (H&E), they were observed under light microscopy and imaged. To perform quantitative analysis on liver tissue sections, three non-overlapping sections in non-consecutive slides were digitized for each individual.

### 2.4. Intestinal Microbiota

At Gs 42, tadpoles from the control, PP, and PS groups were randomly sampled and anesthetized (MS-222) for intestinal microbiota analysis. Because the metamorphosis rate of PE-treated tadpoles was too low, no follow-up experiments could be performed. Anesthetized tadpoles were dissected and their intestines isolated. Tissues were immediately placed in liquid nitrogen for quick freezing and then stored at −80 °C. For each treatment, the intestines of five tadpoles were combined into a single sample. Regrettably, one sample from the control group failed due to technical issues, while the PS group only retained three viable replicates owing to abnormally low metamorphosis rates. Finally, there were 4, 5, and 3 samples for the control group, PP group, and PS group, respectively, to carry out the following experiments. DNA was extracted from samples using an EZNA Fecal DNA Extraction Kit (D4015-01, Omega Bio-Tek, Norcross, GA, USA) following manufacturer’s instructions. The DNA concentration and purity were tested on 1% agarose gels. Purified samples were sent to Novogene Bioinformatics Technology Co., Ltd., (Beijing, China) where the V3–V4 hypervariable region of the 16S rDNA gene was amplified by PCR and Illumina NovaSeq sequencing.

### 2.5. Liver Transcriptome

Due to the low metamorphosis rate of the PS group, four tadpoles at Gs 42 per treatment were randomly collected and anesthetized (MS-222). After dissecting and isolating the livers, the tissues from 4 tadpoles were pooled together as a single sample (n = 1/treatment) for the following experiments. RNA was extracted using a UNIQ-10 column Trizol total RNA extraction kit (B511321, Sangon Biotech, Shanghai, China). The RNA quality was measured using a Nano-600 ultramicro nucleic acid protein analyzer; the RNA integrity was confirmed by 1% agarose gel electrophoresis. Qualified samples were frozen in liquid nitrogen and stored at −80 °C, with three replicates per treatment. After RNA collection, it was sent to Novogene Bioinformatics Technology Co., Ltd. for RNA-Seq analysis. For reference-free genome species such as *R. zhenhaiensis*, transcriptome analysis can be performed by eukaryotic reference-free transcriptome sequencing. RNA-Seq analysis was performed in accordance with the manufacturer’s instructions. mRNA was separated from total RNA, and enriched mRNA was randomly broken into small fragments of approximately 300 bp. Single-stranded cDNA was synthesized using RNA fragments, reverse transcriptase, and random hexamers; cDNA with stable double-stranded structure was synthesized. After PCR amplification and purification, the enriched cDNA was amplified, and a library was established for sequencing.

To evaluate gene expression levels, the Fragments Per Kilobase of transcript per Million mapped reads (FPKM) were obtained using RSEM (V1.2.15) software. Genes that exhibited differential expression between the control and MP exposure groups were identified when the false discovery rate (FDR) was less than 0.01 and the absolute fold change (|FC|) was greater than 2. Subsequently, functional annotation and enrichment analyses for the differentially expressed genes (DEGs) were conducted with the GO and KEGG pathways. Genes related to oxidative stress, energy metabolism, immune inflammation, and apoptosis were selected, and the transcriptional levels of these genes were cluster-heat-mapped. The specific genes associated with each biological process are shown in the Supplementary Materials Table S1.

### 2.6. Statistical Analysis

All experimental values were analyzed using IBM SPSS 23 (IBM, Chicago, IL, USA). Mortality, metamorphosis rate, body weight, body length, and hind limb length are expressed as the mean ± standard deviation (mean ± SD). Kolmogorov–Smirnov, Student’s t, and Levene’s tests were performed to check for normality, independence, and variance homogeneity of the data, respectively, before multiple comparisons using the Games–Howell method were performed. Data were normally distributed, independent of each other, and conformed to the hypothesis test for homogeneity of variance (Kolmogorov–Smirnov test, *p* > 0.05; *t*-test, *p* < 0.05; Lewen test, Sig > 0.05). The chi-square test was employed to calculate statistical mortality rates. One-way analysis of variance (ANOVA) and least significant difference (LSD) tests were used to compare differences in the metamorphosis rate, body weight, and body and hind limb lengths. In addition, we performed a principal component analysis (PCA) to correlate the growth and development indicator variables with gene expression variables. Growth and development index variables were analyzed by the mean value, and gene expression levels were analyzed by FPKM values. Values for all variables were first standardized with the z-score (Z = (x − μ)/σ, where x is the individual observation, μ is the population mean, and σ is the population standard deviation), and then PCA was performed. OriginPro 2017 (OriginLab, Northampton, MA, USA) and Photoshop (Adobe Photoshop CC 2020) software were used to prepare images. The significance threshold was *p* ≤ 0.05.

## 3. Results

### 3.1. Effects of MP Exposure on Mortality, Metamorphosis, and Morphology of Tadpoles

The dechlorinated tap water exhibited the following characteristics: dissolved oxygen content: 7.05 ± 0.56 mg/L; pH: 6.75 ± 0.22; electrical conductivity: 87.11 ± 11.99 μS/cm; total dissolved solids: 43.56 ± 5.96 mg/L; and salinity: 0.04 ± 0.01.

**Figure 1:**
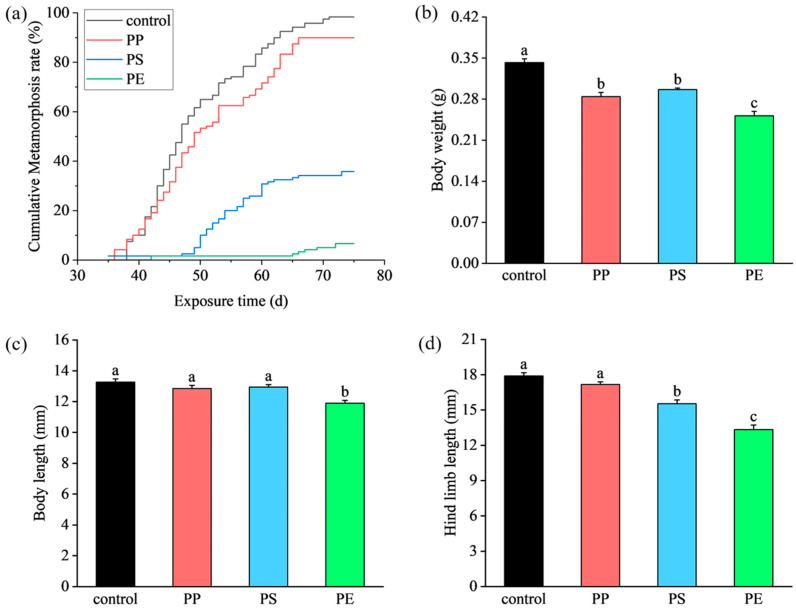
Cumulative metamorphosis curve (**a**), body weight (**b**), body length (**c**), and hind limb length (**d**) in each treatment; different lowercase letters indicate significant differences (*p* < 0.05) (a > b > c).

The mortality rates of the control group, PP group, PS group, and PE group were 1.67 ± 0.83%, 4.17 ± 0.83%, 21.67 ± 3.00%, and 32.50 ± 2.89%, respectively. Exposure to PS and PE significantly increased the mortality rates of tadpoles (*p* < 0.05). During the chronic exposure experiment, the cumulative metamorphosis rate curves of tadpoles in the PP treatment and control group were similar: metamorphosis commenced after 35 d, and the number of metamorphosed tadpoles steadily increased (a metamorphosis rate of approximately 90%). In the PS treatment, metamorphosis began after 45 days. The number of individuals decreased over time, resulting in a final metamorphosis rate of 35.83%. In the PE treatment, metamorphosis did not occur until day 65, even fewer individuals metamorphosed each day, and the metamorphosis rate was 6.67% (Figure 1a). Additionally, body weight in each treatment differed significantly from the control (*p* < 0.05) (Figure 1b). Body length in the PE treatment (left) differed significantly from the control, as did hind limb length in the PE and PS treatments (right) (*p* < 0.05) (Figure 1c,d).

### 3.2. Effects of MP Exposure on Liver Histology

**Figure 2:**
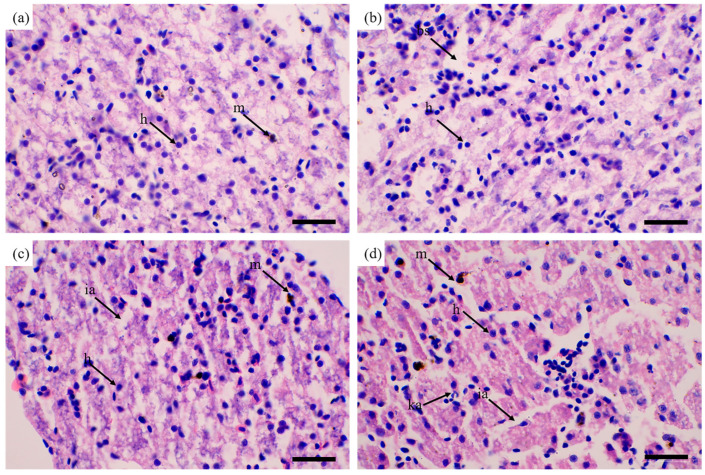
Effect of MPs on tadpole livers. Control group (**a**); PP-treated liver (**b**); PS-treated liver (**c**); PE-treated liver (**d**); h, liver cells; m, pigment cells; bs, blood sinus; ia, intercellular space; ka, nuclear shrinkage. Scale bar 100 μm.

Liver cells of control group tadpoles were uniformly arranged and compact, with few pigment cells (Figure 2a). Qualitative histological assessment revealed no observable structural differences between control and PP-treated livers across three representative tissue regions (Figure 2b). PS-treated livers had greater intercellular spaces (ia) and more pigment cells (Figure 2c). Intercellular spaces in PE-treated livers were obviously enlarged, and even more pigment cells were present (Figure 2d).

### 3.3. Effects of MP Exposure on Intestinal Microbiota

**Figure 3:**
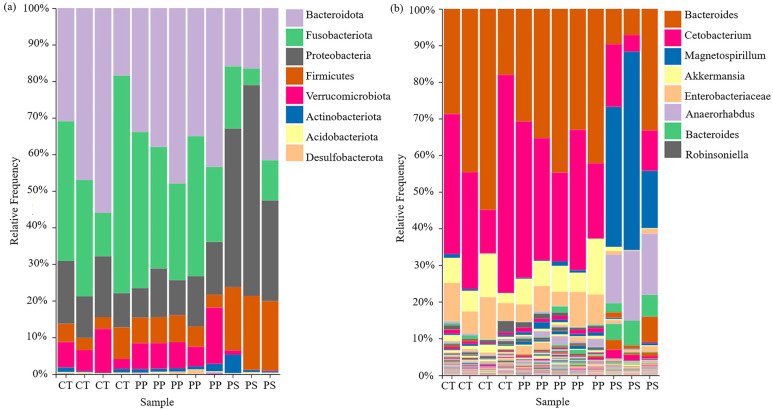
Tadpole intestinal microbial communities after exposure to MPs: (**a**) phyla and (**b**) genera. The vertical axis represents species abundance, and the horizontal axis represents different treatment groups. The color legend represents the species name.

At the phylum level, Bacteroidota, Fusobacteriota, and Proteobacteria were most abundant. The control and PP-treated abundances of these phyla did not differ significantly; compared with the control, the abundance of Bacteroidota in the PS treatment was lower, that of Fusobacteriota decreased significantly, and that of Proteobacteria increased significantly. Additionally, compared with the control and PP treatment, the Firmicutes abundance in the PS treatment increased significantly, and that of Verrucomicrobiota decreased to almost none (Figure 3a). Among the genera, *Bacteroides*, *Cetobacterium*, and *Magnetospirillum* were most abundant. The abundances of each genus did not differ significantly between the control and PP treatment. Compared with the control, the abundances of *Bacteroides* and *Lactobacillus* in the PS treatment were significantly lower, and the abundance of *Magnetospirillum* was significantly higher (Figure 3b).

### 3.4. Correlation Analysis and Identification of Differentially Expressed Genes (DEGs)

**Figure 4:**
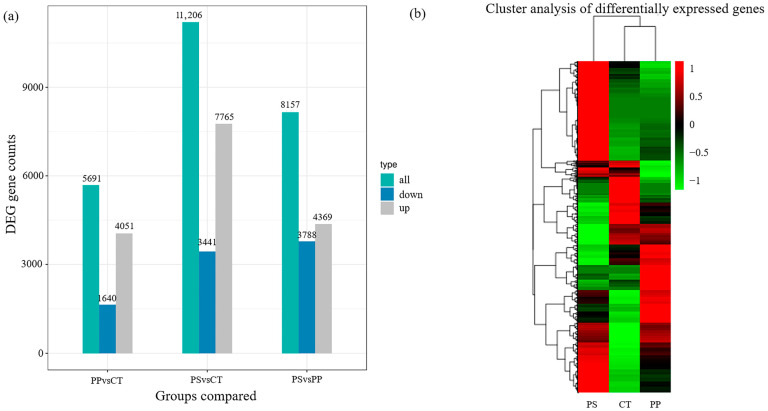
Number of differentially expressed genes in tadpole liver after MP treatment (**a**) and cluster heatmap of differentially expressed genes (**b**).

Transcriptome sequencing was performed on liver tissues of Gs 42 tadpoles following exposure to MPs. Among the three comparison combinations, the PS treatment and control had the largest number (11,206) of DEGs, of which 7765 were up-regulated and 3441 were down-regulated. Compared with the control, there were 5891 DEGs in the PP treatment, of which 4251 were up-regulated and 1640 were down-regulated. Compared with the PP treatment, there were 8157 DEGs in the PS treatment, of which 4369 were up-regulated and 3788 were down-regulated. Compared with the control, most DEGs in the PP and PS treatments were up-regulated (Figure 4a). Clustering heat maps of DEGs also revealed the PP treatment and control to be similar (Figure 4b).

### 3.5. Transcriptional Expression Profiles of Genes

**Figure 5:**
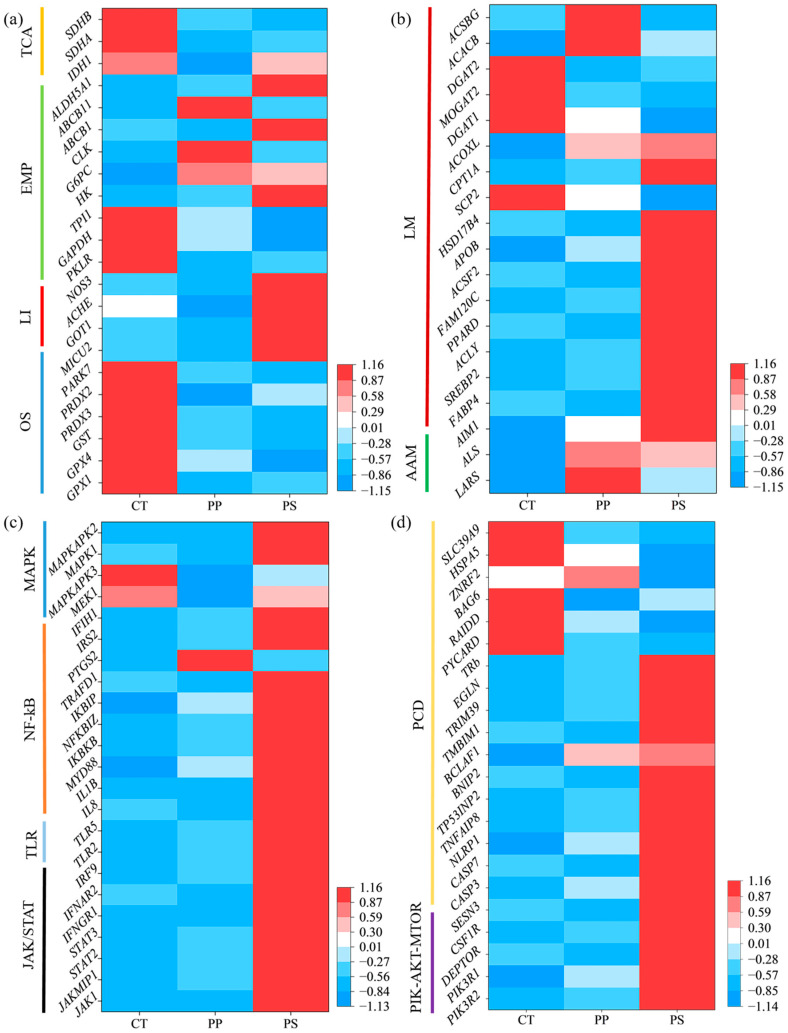
Heat maps of gene expression involved in oxidative stress (OS), liver injury (LI), glycolysis (EMP), and tricarboxylic acid cycle (TCA) (**a**); Amino acid metabolism (AAM) and lipid metabolism (LM) (**b**); JAK/STAT, TLR, NF-κB, and MAPK signaling pathways (**c**); PIK-AKT-MTOR signaling pathway and cell apoptosis (PCD) (**d**). The vertical axis represents different genes, and the horizontal axis represents treatments. The square color represents the expression level of the above genes (FPKM) (red, high; blue, low).

Compared with the control, the expression of the *GPX1*, *GPX4*, *GST*, *PARK7* and *PRDX3* genes related to oxidative stress (OS) was significantly decreased with the PS treatment, and the expression of the *MICU2* gene was significantly increased (*p* < 0.05). The expression of *PRDX2* in the PP treatment decreased significantly (*p* < 0.05). Compared with the control, the levels of *ACHE* were significantly lower in the PP treatment; in the PS treatment, the levels of *GOT1* and *NOS3* were significantly increased (*p* < 0.05) (Figure 5a).

Key genes in glycolysis (EMP) and the tricarboxylic acid cycle (TCA) also changed significantly. For example, the expression levels of *PKLR* and *SDHA* were significantly lower in both PP and PS treatments compared with the control. The levels of *GAPDH*, *TPI1*, and *SDHB* in the PS treatment were significantly lower; the levels of *IDH1* were only significantly lower in the PP treatment (*p* < 0.05). The levels of *G6PC* were significantly higher in both the PP and PS treatments, and *HK*, *ABCB1*, and *ALDH5A1* were significantly higher in the PS treatment. The levels of *CLK* and *ABCB11* were significantly higher in the PP treatment (*p* < 0.05) (Figure 5a). Compared with control levels, the expression of genes related to amino acid metabolism (AAM) was significantly higher in the PP treatment (*p* < 0.05). The levels of many genes related to lipid metabolism (LM) (e.g., *FABP4*, *SREBP2*, *ACLY*, *PPARD*, *FAM120C*, *ACSF2*, *APOB*, *HSD17B4*, *CPT1A*, and *ACOXL*) were significantly higher in the PS treatment. However, the levels of *SCP2*, *DGAT1*, and *MOGAT2* in the PS treatment were significantly lower (*p* < 0.05). The expression levels of *FABP4* and *DGAT2* in the PP treatment were significantly lower, while the expression levels of *ACACB*, *ACSBG,* and *ACOXL* were significantly higher (*p* < 0.05) (Figure 5b).

The expression levels of genes associated with the PI3K-AKT-MTOR, JAK/STAT, and TLR signaling pathways were significantly up-regulated in the PS treatment group compared to the control group (*p* < 0.05) (Figure 5c,d). The expression of genes related to the MAPK signaling pathway such as *MAPK1* and *MAPKAPK2* was also significantly higher in the PS treatment (*p* < 0.05). The expression levels of *MEK1* and *MAPKAPK3* in the PP treatment were significantly lower (*p* < 0.05) (Figure 5c).

*IL1B*, *IL8*, *NFKBIZ*, *IKBKB*, *IKBIP*, *MYD88*, *TRAFD1*, *IRS2*, and *IFIH1* are genes associated with the NF-κB signaling pathway. Their expression levels were significantly higher in the PS treatment (*p* < 0.05). The expression levels of *NFKBIZ* and *PTGS2* in the PP treatment were also significantly higher (*p* < 0.05) (Figure 5c). The apoptosis-related genes *CASP3*, *CASP7*, *NLRP1*, *TNFAIP8*, *TP53INP2*, *BNIP2*, *BCLAF1*, *TMBIM1*, *TRIM39*, *EGLN*, and *TRB* were significantly higher in the PS treatment (*p* < 0.05), while *PYCARD*, *RAIDD*, *ZNRF2*, *HSPA5*, and *SLC39A9* showed significantly lower levels (*p* < 0.05). Compared with the control group, the levels of the *BAG6* and *PYCARD* genes in the PP treatment were significantly lower (*p* < 0.05) (Figure 5d).

**Figure 6:**
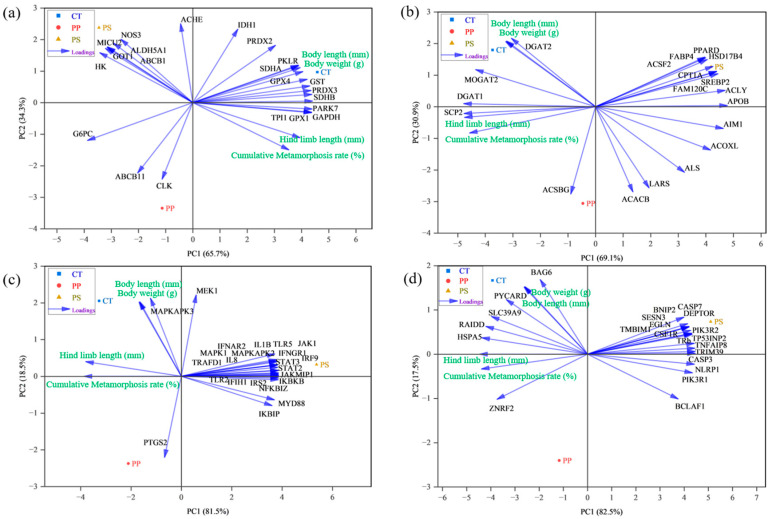
Principal component analysis (PCA) of growth and development indices and related genes in the liver tissue of *Rana zhenhaiensis* tadpoles after exposure to polypropylene (PP) and polystyrene (PS). Panel (**a**) displays oxidative stress (OS)-, liver injury (LI)-, glycolysis (EMP)-, and tricarboxylic acid cycle (TCA)-related genes alongside growth and development indicators. Panel (**b**) shows amino acid metabolism (AAM) and lipid metabolism (LM) genes, as well as growth and development indicators. Panel (**c**) includes genes from the JAK/STAT, TLR, NF-κB, and MAPK signaling pathways along with growth and development indicators. Panel (**d**) illustrates the PIK-AKT-MTOR signaling pathway and apoptosis (PCD) in relation to growth and development indicators. The horizontal axis represents the first principal component (PC1), while the vertical axis represents the second principal component (PC2). Growth indicators include body weight, body length, hind limb length, and cumulative metamorphosis rate (indicated by green labels). Black tags denote different genes, and blue arrows indicate the contributions and directions of various original variables.

The cumulative contribution of the first principal component (PC1) and the second principal component (PC2) is 100% (Figure 6a). In the positive direction of PC1, the *PARK7* and *GAPDH* genes had the greatest contributions, while in the opposite direction, the *G6PC* and *HK* genes contributed the most. For PC2, *ACHE* and *IDH1* genes had the highest contributions in the positive direction, whereas the *CLK* and *ABCB11* genes, along with the cumulative metamorphosis curve, contributed the most in the negative direction (Figure 6a). In the PP group, EMP-related genes (*G6PC*, *CLK*, *ABCB11*) showed negative correlations with tadpole body weight and body length. Similarly, in the PS group, OS-related (*MICU2*), LI-related *(NO3*, *GOT1*), and EMP-related genes (*HK*, *ALDH5A*, *ABCB1*) were negatively correlated with the metamorphosis rate and hind limb length (Figure 6a).

In Figure 6b, the cumulative contribution of PC1 and PC2 also totals 100%. Here, the *APOB* and *ACLY* genes had the most significant contributions in the positive direction of PC1, while the *DGAT2* gene and body length contributed the most in the positive direction of PC2. In the PS group, numerous LM-related genes also exhibited negative correlations with the tadpole metamorphosis rate and hind limb length (Figure 6b). Figure 6c shows that the contribution of PC1 is 81.5%. In the positive direction of PC1, the *STAT3*, *IKBKB*, *JAKMIP1*, and *NFKBIZ* genes contributed the most, while the cumulative metamorphosis curve and hind limb length had the greatest contributions in the negative direction. Similarly, Figure 6d indicates that the contribution of PC1 is 82.5%. In the positive direction of PC1, the *CASP3*, *TRIM39*, *TRb*, and *NLRP1* genes contributed the most. In contrast, hind limb length and the cumulative metamorphosis curve contributed the most in the negative direction. In the PS group, most immune response and PCD genes were negatively correlated with tadpole metamorphosis rate and hind limb length (Figure 6c,d). In conclusion, the PCA analysis indicates that the growth and development of *R. zhenhaiensis* tadpoles are strongly correlated with various genes related to biological processes.

## 4. Discussion

### 4.1. Effects of MPs on Mortality, Metamorphosis Rate, and Morphology of Tadpoles

The mortality rate serves as the most direct indicator of toxicity. In this study, both PE and PS significantly increased mortality for tadpoles, suggesting that they exhibit greater toxicity compared to PP. In addition, amphibian metamorphosis rate and body size are commonly used to assess the toxicological capacity and sensitivity of chemical substances [28]. We report that the cumulative metamorphosis rates of tadpoles in the PP treatment and control groups are similar, but they are significantly lower in the PS and PE treatments compared with the control, and metamorphosis is delayed. This indicates that PP exposure did not significantly affect tadpole metamorphosis, whereas PS and PE exposure did, specifically delaying it. In addition, exposure to PP, PS, and PE significantly reduced tadpoles’ body weight, with PS and PE additionally impairing hind limb development. Notably, PE uniquely significantly decreased the body length of tadpoles. Compared with control tadpoles, we report the effects of PP MPs on tadpole growth and development to be less than those of PE and PS MPs.

Studies have demonstrated that exposure to MPs can affect amphibian growth and development [29]. For example, MP ingestion severely affected the growth and survival of Lataste’s frog (*Rana latastei*) tadpoles, whose body weight decreased significantly with increased MP density (from 1–50 mg/L) [30]. Exposure to 60 mg/L PE MPs significantly shortened the body and head lengths of *P. cuvieri* tadpoles [20], and 1800 particles/mL PS MPs negatively affected the survival of the common midwife toad (*Alytes obstetricans*) tadpoles, whose growth and body condition decreased with increasing concentration [31]. In toxicological studies of MPs, two primary forms are commonly utilized: commercially available microspheres [13,23] and mechanically fragmented microplastics with diverse shapes [21,22]. Microspheres serve as a more controllable experimental material for toxicological testing due to their standardized parameters such as size, shape, and surface properties, while fragmented microplastics better mimic environmentally relevant conditions owing to their irregular morphologies and heterogeneous compositions that closely resemble real-world plastic pollution. Therefore, the actual MP threats faced by tadpoles in natural environments may differ from the results in this study. However, fortunately, the MP concentrations used in laboratory settings are likely uncommon in real-world scenarios, suggesting that the ecological risks may not be as high as suggested by laboratory findings.

### 4.2. PE and PS Exposure Damaged the Liver Tissue of Tadpoles

The liver is an important metabolic organ that mediates the absorption, digestion, synthesis, and storage of various biochemical components; detoxifies environmental pollutants; and plays a key role in maintaining biological energy homeostasis [32]. It is the center of lipid metabolism and bile salt secretion [33]. Changes to liver histopathology can indicate adverse effects of MPs on its function. Common pathological features include hepatic nuclear atrophy, sinus congestion, and abnormal enlargement of intercellular spaces. We report (compared with control group tadpoles) enlargement of liver intercellular spaces and increased numbers of pigment cells with PE and PS treatments. PS MPs can cause degenerative necrosis and inflammation in the liver of American bullfrog (*Rana catesbeiana*) tadpoles. Liver cell vacuolation and loose cell arrangement can be observed in sections [34]. Following exposure to PE MPs, the liver of *P. cuvieri* tadpoles was damaged, showing vasodilation, hypertrophy, and hyperplasia, while cell nuclei had a greater area, circumference, volume, and longer long and short axis lengths [25]. Liver vacuolation may be related to energy expenditure and protein synthesis inhibition in response to chemical stimulation [35]. The increase in melanin macrophage centers in the liver is also associated with phagocytosis activity during liver detoxification [36]. Therefore, the changes that we report in liver histopathology (expansion of intercellular spaces) with PE and PS treatments may reduce liver function, explaining the decreased tadpole growth and developmental delay.

### 4.3. PS Exposure Induced Dysregulation of Intestinal Microbiota of Tadpoles

Previous studies from our group and others have demonstrated that MPs mainly accumulate in tadpole gills and the gastrointestinal tract [20,23]. Thus, the intestinal microbiota can be directly influenced by exogenous substances such as MPs. The intestinal microbiota are important for the maintenance of the gut barrier function. Because of their sensitivity, the composition, structure, and function of intestinal microbes are important in ecotoxicological assessment [37]. We found that the intestinal microbiota community structure and abundance in the PP treatment and control were similar, but significant changes occurred in the PS treatment (because so few tadpoles were available, no results were obtained for the PE treatment). Proteobacteria, Firmicutes, Bacteroidota, and Fusobacteriota dominate the intestinal microbiota of tadpoles [38]. An increase in the relative abundance of Proteobacteria may indicate intestinal dysbiosis. A stable ratio of Bacteroidota to Firmicutes may play an important role in maintaining intestinal microbiota homeostasis [39]. Therefore, an increase in the abundance of Proteobacteria and a decrease in the ratio of Bacteroidota to Firmicutes in the PS treatment may reflect metabolic dysfunction in intestinal microbial communities and possible damage to the tadpole immune system. A decrease in Bacteroidota is associated with inflammatory bowel disease, ulcerative colitis, and cancer [40]. A decrease in Fusobacteriota can cause infectious diseases and the prevalence of diseases with tissue necrosis [38], whereas *Cetobacterium* reduces infectious diseases and restores immune homeostasis [41]. However, the abundances of Bacteroidota, Fusobacteriota, and *Cetobacterium* in the PS treatment decreased significantly, damaging intestinal microecology, affecting the intestinal mucosal immune response, and reducing the digestion and absorption capacity. Considerable evidence indicates that changes in the intestinal microbiome usually lead to dysregulation of the microbiome, leading to intestinal diseases and metabolic disorders [32]. Therefore, we infer that changes in the intestinal microbiota of tadpoles with the PS treatment obstructed metabolic activities and ultimately negatively affected tadpole growth and their ability to move.

### 4.4. PP and PS Exposure Altered Transcription Levels of Liver Genes in Tadpoles

*GPX* and *PRDX* are antioxidant genes that can specifically remove intracellular superoxide anions, and by reducing the level of intracellular ROS, they can contribute to avoiding damage to the body. *GST* is the main detoxification gene for anti-injury and anticancer transformation of cells in vivo and participates in the biotransformation of exogenous and endogenous toxic substances [42]. *PARK* acts as a sensor for oxidative stress and apparently protects neurons from oxidative stress and cell death. PS MPs can accumulate in grass carp (*Ctenopharyngodon idella*) and induce oxidative stress in its liver, and its metabolic disorders and oxidative stress can promote each other and even exacerbate organ damage [43]. We demonstrate that PS MPs can lead to impaired antioxidant capacity by significantly reducing the mRNA levels of *GPX1*, *GPX4*, *PRDX2*, *PRDX3*, *GST*, and *PARK7*. This can trigger the overproduction of ROS and induce oxidative stress in the body [44].

The tricarboxylic acid cycle is the main pathway of energy metabolism. As key enzymes in this pathway, the expression levels of the *IDH*, *SDHA*, and *SDHD* genes are inhibited in the PP and PS treatments, possibly indicating that the body’s energy metabolism is reduced following exposure to MPs [45]. Glycolysis is the main pathway of carbohydrate breakdown in eukaryotes, and *GAPDH* and *TPI1* are essential for glycolysis [46]. Proteins encoded by *HK*, *G6PC*, and *PKLR* are rate-limiting enzymes that catalyze the first and last irreversible steps of glycolysis and gluconeogenesis, maintaining glucose balance during oxidative stress [47]. *CLK* is a mitochondrial hydroxylase that is necessary for the biosynthesis of ubiquinone (Coenzyme Q or UQ) [48]. *ABCB1* and *ABCB11* are a large class of membrane protein genes that can mediate a wide range of transport functions. Glycolysis-related genes and the upregulation of *ABCB1* may be related to glycolytic activation under MP exposure [49]. With the PS treatment, the expression of *GAPDH*, *TPI1*, and *PKLR* decreased, while the expression of *HK*, *G6PC*, *CLK1*, *ABCB1*, and *ABCB11* increased, possibly disrupting the glucose balance and inducing disorder in liver glucose metabolism. Genes such as PPAR family genes, *DGAT*, *MOGAT2*, *ACLY*, *ACACB*, and *ACSBG* also regulate fat production. We report that the expression of such genes trends downward in both the PP and PS treatments. *FABP4* and *APOB* regulate lipid transport, and *CPTLA*, *SCP2*, *HSD17B4*, and *ACOXL* are genes related to fatty acid β oxidation [33,50]. *SREBP2* specifically regulates cholesterol and fatty acid metabolism to maintain lipid homeostasis [51]. Disruption of these genes indicates abnormalities in lipid metabolism after exposure to two types of MPs. Additionally, *AIM1*, *LARS*, and *ALS* trended upwards in both MP treatments, indicating that MPs promoted amino acid catabolism in the body [52]. This further confirms that MP exposure can simultaneously affect carbon, lipid, and amino acid metabolism and indicates that the mechanism of liver toxicity induced by PP or PS MPs is complex.

Many studies have reported that MPs enter cells and initially generate oxidative stress via reactive oxygen species (ROS), which then trigger various biological responses such as oxidative stress-induced signaling pathways and immune inflammation [53]. Interferon is a multifaceted protein that plays a key role in coordinating a powerful antiviral immune response and regulating the complex host immune landscape. The main signaling pathway for interferon activation is the JAK/STAT signaling pathway [54]—a key signal transduction cascade that regulates important biological responses such as development, immunity, and tumorigenesis [55]. *MYD88* is a key connector molecule that connects JAK/STAT with the MAPK signaling pathway [56], which plays an important role in cell response to extracellular stimuli and influences immune response and apoptosis [57]. *MDA5* belongs to the RIG-I receptor family and is involved in innate immunity. An antiviral response can be generated during viral infection by identifying ligands that activate interferons [58]. We report that PS MP exposure significantly upregulates the expression of genes involved in MAPK, JAK/STAT, and the immune response, similar to the results reported for the liver of *D. rerio* [59]. Additionally, TLR is the host’s first line of defense against invading pathogens. PS MPs target binding to *TLR2* and promote an inflammatory response by activating the NF-κB signaling [60]. For these pathway genes, we report that inflammatory cytokines or chemokines (e.g., *IL1B* and *IL8* genes) are significantly up-regulated and that the TLR and NF-κB signaling pathways are significantly enriched. Accordingly, we speculate that PS MPs can induce hepatotoxicity by activating innate immune cells and the inflammatory response.

After MPs activate the NF-κB pathway, the BCL2 and BAX proteins participate in the regulation of apoptosis through the mitochondrial pathway, and BAX activates the downstream apoptosis executive protein CASP3, ultimately leading to apoptosis [61]. Additionally, proteolytic maturation encoded by *CASP7* can participate in apoptosis and inflammation [62]. *BCLAF1* is a pro-apoptotic transcription factor that plays an important role in interfering with apoptotic pathways [63]. TP53INP2 is an important part of autophagosome formation and makes cells sensitive to apoptosis [64]. *TNFAIP3* genes involved in other pathways also play roles in apoptosis [65]. Our results suggest that PS MPs may induce apoptosis by changing the expression levels of these genes. In the PP treatment, the expression levels of *MEK1*, *MAPKAPK3*, *BAG6*, and *PYCARD* were significantly down-regulated, while those of *NFKBIZ*, *IFIH1*, *PTGS2*, and *PIK3R1* were significantly up-regulated. This may indicate that PP MPs affect the immune response and apoptosis of tadpoles. We further speculate that the effects of PS MPs on tadpoles are more negative than those of PP MPs. In general, MPs can accelerate cell apoptosis by mediating the JAK/STAT, MAPK, TLR, and NF-κB signaling pathways, leading to decreased immunity. In doing so, they cause irreversible, negative effects on body growth and survival.

## 5. Conclusions

We conclude that different MPs affect the growth and development of *R. zhenhaiensis* tadpoles in different ways, but the negative effects of PE and PS MPs exceed those of PP. Additionally, MPs induce oxidative stress, metabolic disorder, immune response, and apoptosis in the liver by interfering with relevant signaling pathways, as well as dysregulating the intestinal microbiota. However, PS and PP exhibited different interference patterns with these signaling pathways. The distinct correlations between the alterations in signaling pathways and morphological changes highlight polymer-specific mechanisms underlying MP-induced growth and development toxicity in tadpoles.

## Data Availability

The data from this study are available upon reasonable request to the corresponding authors. The raw data of intestinal microbiota and liver transcriptome have been deposited into CNGB Genome Sequence Archive with accession numbers CRA023047 and CRA023057, respectively.

## Supplementary Materials

The following supporting information can be downloaded at: https://www.mdpi.com/article/10.3390/toxics13030165/s1, Table S1: Gene list related to signaling pathway.
