# Supplementary material for: Effects of Exposure to Different Types of Microplastics on the Growth and Development of Rana zhenhaiensis Tadpoles
Source: Toxics. 2025 Feb 26;13(3):165. doi: 10.3390/toxics13030165 (PMC11945619; doi:10.3390/toxics13030165)
Supplement: Supplementary file 1 [file toxics-13-00165-s001.zip › toxics-3484252-supplementary.pdf]

Table S1 Gene list related to biological process

**biological process**	**gene**
Oxidative stress (OS)	glutathione peroxidase 1 (*GPX1*), *GPX4*, Glutathione S-transferase (*GST*), Parkinsonism associated deglycase (*PARK7*), Peroxiredoxin 2 (*PRDX2*), *PRDX3*, mitochondrial calcium uptake 2 (*MICU2*)
Liver injury (LI)	glutamic-oxaloacetic transaminase 1 (*GOT1*), acetylcholinesterase (*ACHE*), nitric oxide synthase 3 (*NOS3*)
Glycolysis (EMP)	pyruvate kinase L/R (*PKLR*), glyceraldehyde-3-phosphate dehydrogenase (*GAPDH*), triosephosphate isomerase 1 (*TPI1*), glucose-6-phosphatase catalytic subunit 1 (*G6PC*), hexokinase (*HK*), ATP binding cassette subfamily B member 1 (*ABCB1*), *ABCB11,* aldehyde dehydrogenase 5 family member A1 (*ALDH5A1*), Cdc-like kinase 2 (*CLK*)
Tricarboxylic acid cycle (TCA)	Succinate dehydrogenase complex flavoprotein subunit A (*SDHA*), *SDHB,* isocitrate dehydrogenase 1 (*IDH1*)
Amino acid metabolism (AAM)	Enoyl-CoA hydratase/isomerase (*AIM1*), leucyl-TRNA synthase 1 (*LARS*), acetyllactate synthase (*ALS*)
Lipid metabolism (LM)	fatty acid binding protein 4 (*FABP4*), sterol regulatory element binding protein (*SREBP2*), ATP citrate lyase (*ACLY*), peroxisome proliferator activating receptor Delta (*PPARD*), Family With Sequence Similarity 120 Member C (*FAM120C*), Acyl-CoA Synthetase Family Member 2 (*ACSF2*), Apolipoprotein b (*APOB*), hydroxysteroid 17-β dehydrogenase 4 (*HSD17B4*), carnitine palmitoyltransferase 1A (*CPT1A*), and acyl-CoA oxidase-like (*ACOXL*), sterol carrier protein 2 (*SCP2*), diacylglycerol o-acyltransferase (*DGAT1*), monoacylglycerol o-acyltransferase 2 (*MOGAT2*), acetyl-CoA carboxylase β (*ACACB*), Acyl-CoA synthetase bubblegum family member (*ACSBG*)
JAK/STAT signaling pathway	Janus Kinase 1 (*JAK1*), Janus Kinase And Microtubule Interacting Protein 1 (*JAKMIP1*), Signal Transducer And Activator Of Transcription 2 (*STAT2*), *STAT3*, interferon α and β receptor subunit 2 (*IFNAR2*), Interferon Gamma Receptor 1 (*IFNGR1*), interferon regulatory factor 9 (*IRF9*)
TLR signaling pathway	Toll-like receptor 2 (*TLR2*), *TLR5*
PIK-AKT-MTOR signaling pathway	phosphoinositol-3-kinase regulatory subunit 1 (*PIK3R1*), *PIK3R2*, DEP domain containing MTOR interacting proteins (*DEPTOR*), Colony Stimulating Factor 1 Receptor (*CSF1R*), sestrin 3 (*SESN3*)

MAPK signaling pathways	mitogen-activated protein kinase 1 (*MAPK1*), MAPK-activated protein kinase 2 (*MAPKAPK2*), *MAPKAPK3*, mitogen-activated protein kinase kinase 1 (*MEK1*)
NF-kB signaling pathway	Interleukin 1β (*IL1B*), *IL8*, Nuclear factor kappa B inhibitor zeta (*NFKBIZ*), Inhibitor of nuclear factor kappa B kinase subunit beta (*IKBKB*), IKBKB interacting protein (*IKBIP*), Myeloid differentiation primary response 88 (*MYD88*), interferon containing a TRAF-type zinc finger domain containing 1 (*TRAFD1*), insulin receptor substrate 2 (*IRS2*), Interferon induced with helicase C domain 1 (*IFIH1*), prostaglandin-endoperoxidase synthase (*PTGS2*)
Cell apoptosis (PCD)	caspase 3 (*CASP3*), *CASP7*, NLR Family Pyrin Domain Containing 1 (*NLRP1*), TNF-α-inducing protein 8 (*TNFAIP8*), tumor protein P53 inducing nuclear protein 2 (*TP53INP2*), BCL2-interacting protein 2 (*BNIP2*), BCL-2 associated transcription factor 1 (*BCLAF1*), transmembrane BAX inhibitor motif containing 1 (*TMBIM1*), Tripartite Motif Containing 39 (*TRIM39*), EGL-9 family hypoxia Inducible factor 2 (*EGLN*), T Cell Receptor Beta Locus (*TRB*), PYD And CARD Domain Containing (*PYCARD*), RIPK1 associated via death domain (*RAIDD*), Zinc And Ring Finger 2 (*ZNRF2*), Heat Shock Protein Family A (Hsp70) Member 5 (*HSPA5*), Solute Carrier Family 39 Member 9 (*SLC39A9*), BAG Cochaperone 6 (*BAG6*)
